# Aspartate Aminotransferase to Platelet Ratio Index (APRI) as a Predictor of Metabolic Syndrome (MetS) Development in Individuals With Type 2 Diabetes Mellitus

**DOI:** 10.7759/cureus.63389

**Published:** 2024-06-28

**Authors:** Amir Jalisi, Rijad Jahić, Avdo Kurtović, Miralem Đešević, Azra Husić-Selimović, Enisa Hodžić, Edina Lazović Salčin, Orhan Lepara, Almir Fajkić

**Affiliations:** 1 Department of Pathophysiology, Faculty of Medicine, University of Sarajevo, Sarajevo, BIH; 2 Department of Internal Medicine and Cardiology, Sarajevo University Clinical Center, Sarajevo, BIH; 3 Department of Surgery, Tuzla University Clinical Center, Tuzla, BIH; 4 Department of Cardiology, Polyclinic Eurofarm Center Sarajevo, Sarajevo, BIH; 5 Department of Internal Medicine, Dr. Abdulah Nakas General Hospital, Sarajevo, BIH; 6 Department of Internal Medicine and Cardiology, Clinic for Heart, Blood Vessel and Rheumatic Diseases, Sarajevo University Clinical Center, Sarajevo, BIH; 7 Department of Pathology, Faculty of Medicine, University of Sarajevo, Sarajevo, BIH; 8 Department of Human Physiology, Faculty of Medicine, University of Sarajevo, Sarajevo, BIH

**Keywords:** aspartate aminotransferase-to-platelet ratio index (apri score), liver fibrosis indices, fatty liver, metabolic syndrome, diabetes mellitus

## Abstract

Introduction: Despite ongoing findings on the relationship between liver fibrosis in nonalcoholic fatty liver disease (NAFLD) and metabolic syndrome (MetS), this association in diabetic patients remains unclear. Early diagnosis of liver fibrosis is important due to the easily available diagnostic tools, such as noninvasive indices that combine clinical and laboratory variables, and the possibility of preventing its complications in type 2 diabetes mellitus (T2DM) patients with MetS.

Objective: This study examines the potential predictive values of non-invasive liver fibrosis indices for MetS in T2DM patients.

Patients and methods: Over the course of a two-year prospective, observational, clinical study, 80 individuals with T2DM randomly selected from the Diabetes Counseling Centers of the Public Institution Health Center of Sarajevo Canton were divided into two groups: T2DM-MetS and T2DM-non-MetS, based on the development of MetS. The study included individuals with T2DM aged 30 to 60 who were clinically diagnosed without MetS, voluntarily agreed to participate, and provided complete data in the collection forms. Serum samples from the patients were assessed for levels of liver enzymes, platelet counts, total cholesterol, high-density lipoprotein cholesterol, fasting glucose, and triglycerides. Various equations were utilized to calculate liver fibrosis indices, including the Aspartate Aminotransferase to Platelet Ratio Index (APRI), Aspartate Aminotransferase to Gamma-Glutamyl Transferase to Platelet Ratio (AGPR), Aspartate Aminotransferase to Alanine Aminotransferase Ratio to Platelet Ratio Index (AARPRI), Fibrosis-4 (FIB-4) Index, Forns Index, and Gamma-Glutamyl Transpeptidase to Platelet Ratio (GPR). Receiver operating characteristic (ROC) analysis was utilized to determine the usefulness of noninvasive liver fibrosis indices for diagnosing MetS in individuals with T2DM. Logistic regression analysis was used to predict the onset of MetS in T2DM patients.

Results: Significant differences in the values of APRI (p<0.001), AGPR (p<0.05), AARPRI (p<0.001), and the FIB-4 index (p=0.001) were observed in T2DM-MetS individuals compared to T2DM-non-MetS. According to ROC analysis, the area under the curve (AUC) was found to be highest for APRI (0.84), followed by FIB-4 (0.783) and AARPRI (0.747). Logistic regression analysis identified APRI as an independent positive predictor of MetS (OR 18.179, 95% CI 6.035-24.58, p=0.015).

Conclusion: This research highlights the effectiveness of the APRI index as a reliable predictor of MetS development in individuals with T2DM.

## Introduction

Metabolic syndrome (MetS) is highly prevalent worldwide and is closely associated with type 2 diabetes mellitus (T2DM), primarily due to the increasing rates of obesity and sedentary lifestyles [1]. The coexistence of MetS and T2DM is common and significantly elevates the risk of chronic complications and mortality. However, the prevalence of MetS among T2DM patients remains contentious due to varying definitions of MetS, highlighting the necessity to identify risk factors and preventive measures for this syndrome in diabetic patients [2].

Obesity, particularly visceral fat accumulation, is intrinsically linked to both MetS and T2DM, resulting in similar adverse health outcomes such as dysregulated blood sugar levels, insulin resistance, abnormal lipid profiles, cardiovascular diseases, atherosclerosis, non-alcoholic fatty liver disease (NAFLD), depression, infertility, and certain cancers [3].

The global obesity epidemic, intensified by Westernized lifestyles and earlier onset, has contributed to an upsurge in NAFLD cases. Between 2014 and 2017, the global prevalence of NAFLD among T2DM patients was 55%, with 37% having non-alcoholic steatohepatitis (NASH) and 17% presenting with advanced fibrosis. T2DM significantly heightens the risk of cirrhosis and fibrosis in multiple organs, including the liver, heart, kidneys, and lungs [4,5].

Visceral fat strongly correlates with NAFLD, establishing a bidirectional relationship between hepatic fatty acid accumulation and MetS - the level of fatty acids in the liver is indirectly linked with MetS, acting as both a cause and a result of the syndrome [6]. Fatty liver produces excess glucose and lipids, especially very low-density lipoproteins (VLDL), which are crucial in MetS. It also raises cardiovascular risk factors such as inflammation and various blood clotting factors. Contributing factors to this relationship include overnutrition and high carbohydrate intake, which exacerbate insulin resistance [7].

Fibrosis, a consequence of advanced NAFLD, predicts poor outcomes and advanced liver fibrosis is often linked to more severe metabolic comorbidities. The incidence of NAFLD-associated fibrosis has more than doubled in the past two decades, resulting in increased mortality, liver cancer, and liver transplantation rates [8]. Despite this, routine liver fibrosis screening for individuals with MetS or T2DM is not universally recommended due to ambiguous benefits and associated costs. Nonetheless, guidelines suggest screening these high-risk groups due to their elevated fibrosis rates [9].

While liver biopsy remains the gold standard for diagnosing NAFLD and associated fibrosis, its invasiveness renders it impractical, particularly in early disease stages. FibroScan is widely used to evaluate liver stiffness and can effectively rule out significant hepatic fibrosis, according to the European Association for the Study of the Liver (EASL) and the American Association for the Study of Liver Diseases (AASLD) recommends it. However, despite being noninvasive, FibroScan is expensive and may not be accessible at all medical facilities [10]. Serum liver enzyme biomarkers offer a non-invasive alternative and could potentially serve as novel candidate biomarkers for MetS and its clinical outcomes. Elevated liver enzyme levels are associated with MetS components and the syndrome itself. However, liver enzymes such as transferases lack specificity for hepatocellular damage, as they are also present in heart muscle, skeletal muscle, kidneys, brain, pancreas, and blood cells. Additionally, many individuals with the full spectrum of NAFLD have normal liver enzyme levels, and their changes do not correspond with changes in the fibrosis stage [11-14].

Given these considerations, it is crucial to explore whether liver function parameters, combined with anthropometric and hematological parameters, can elucidate the complex relationship between NAFLD, liver fibrosis, and MetS, particularly in T2DM patients. This study aims to evaluate the potential role of non-invasive liver fibrosis indices as predictors of MetS development in T2DM.

## Materials and methods

Study sample and design

The research was designed as a prospective, observational, clinical study spanning 24 months. It initially enrolled 94 participants with T2DM, 48 males and 46 females, all without MetS, randomly selected from the Diabetes Counseling Centers of the Public Institution Health Center of Sarajevo Canton. After the observation period, data from 80 individuals who met the study's criteria were subjected to statistical analysis.

Inclusion criteria

The study initially included individuals with T2DM aged 30 to 60 who were clinically diagnosed without MetS, voluntarily agreed to participate, and provided complete data in the collection forms.

Exclusion criteria

Participants with T2DM were excluded from the study if they had any of these conditions: acute or chronic liver diseases, cardiovascular or kidney diseases, cancers, or chronic alcohol consumption. Moreover, individuals with aminotransferase levels exceeding five times the upper normal limit, those on medications that influence serum liver enzyme levels and platelet counts (PLT), or those with incomplete data in their collection forms were also excluded.

Upon completing the 24-month observation period, participants with T2DM who met the study's criteria were sorted into two distinct groups: T2DM-MetS, consisting of individuals who met the MetS criteria (n=48), and T2DM-non-MetS, consisting of individuals who did not meet the MetS criteria (n=32).

Prior to participation, all T2DM participants were thoroughly informed about the study's aims and procedures, and they gave their written informed consent. The study adhered to the ethical guidelines outlined in the Declaration of Helsinki regarding patients' rights in biomedical research (2013 revision) and received ethical approval from the Ethical Committee of the University of Sarajevo - Faculty of Medicine (approval number 02-3-1-4747).

Definition of MetS

MetS was diagnosed if at least three of the following five criteria, as recommended by the National Cholesterol Education Program (NCEP) and Adult Treatment Panel III (ATP III), were met: central obesity (waist circumference (WC): >102 cm for men, >88 cm for women), triglycerides (≥1.7 mmol/L), high-density lipoprotein (HDL) cholesterol (≤1.03 mmol/L for men, ≤1.29 mmol/L for women), blood pressure (≥139/≥89 mm Hg), and fasting glucose (≥6.1 mmol/L) [15].

Data collection

At the beginning and conclusion of the study, detailed data were collected from all T2DM participants, including demographics, medical history, height, weight (for BMI calculation), WC, and blood pressure. Blood samples were taken from the cubital vein for laboratory analysis, with serum separated by centrifugation within 30 minutes and stored at -20°C. Tests performed included total cholesterol, triglycerides, HDL cholesterol, fasting glucose, PLT, and liver enzymes (aspartate aminotransferase (AST), alanine aminotransferase (ALT), alkaline phosphatase (AP), gamma-glutamyl transferase (GGT)). Biochemical tests were conducted using standard enzymatic colorimetric techniques (Dimension RxL Max, Dade Behring, Germany). PLTs were determined using a Beckman Coulter STKS Hematology Analyzer (Beckman Coulter, Inc., Brea, USA), and liver enzyme levels were assessed spectrophotometrically with a SELECTRA analyzer (ELITechGroup, Puteaux, France).

Based on the obtained values, the following noninvasive indices of liver fibrosis were calculated using appropriate formulas [16,17]:

$APRI=\left(AST\left(U/L\right)/{ULN}^\ast\right)/Plt\left({10}^9/L\right)\times100$

$AGPR=\left(AP\left(U/L\right)\right)+\left(GGT\left(U/L\right)\right)/Plt\left({10}^9/L\right)$

$FIB-4\ index=Age\left(years\right)\times AST\left(U/L\right)/\left[Plt\ \left({10}^9/L\right)\times A L T\left(U/L\right)1/2\right]$

$AARPRI=\left(AST/ALT\right)/Plt\left({10}^9/L\right)/150$

$FORNS\ index=7.811-3.131\times ln\left[Plt\left({10}^9\right)\right]\times0.781\times ln\left[GGT\left(U/L\right)\right]+3.467\times ln\left[age\left(years\right)\right]-0.014\left[total\ cholesterol\left(mg/dl\right)\right]$

$GPR=\left(GGT\left(U/L\right)/{ULN}^{\ast\ast}\right)/Plt\left({10}^9/L\right)\times100$

* ULN (upper limits of normal) for AST < 40 U/L

** ULN for GGT < 73 U/L

AP: alkaline phosphatase; AARPRI: aspartate aminotransferase to alanine aminotransferase ratio to platelet ratio index; AGPR: aspartate aminotransferase to gamma-glutamyl transferase to platelet ratio; ALT: alanine aminotransferase; APRI: aspartate aminotransferase to platelet ratio index; AST: aspartate aminotransferase; FIB-4: Fibrosis-4 index; FORNS index: Forns index; GGT: gamma-glutamyl transferase; GPR: gamma-glutamyl transpeptidase to platelet ratio; PLT: platelets

Data analysis

Statistical analysis was performed using MS Excel (2010; Microsoft® Corp., Redmond, USA) and Statistical Package for the Social Sciences (IBM SPSS Statistics for Windows, IBM Corp., Version 22.0, Armonk, NY). The Shapiro-Wilk test checked the normality of variable distribution. For normally distributed continuous variables, the mean (X) and standard deviation (SD) were calculated; for non-normally distributed variables, the median and interquartile range were used. The student's t-test assessed differences in normally distributed variables, while the Mann-Whitney U test was applied for non-normally distributed variables. Receiver operating characteristic (ROC) curves and the area under the curve (AUC) were utilized to identify the best biomarker cut-off values for distinguishing MetS from non-MetS individuals, with accuracy evaluated within a 95% confidence interval (CI). Logistic regression predicted the onset of MetS in individuals with T2DM. A p-value of <0.05 was considered statistically significant.

## Results

Table 1 presents the mean values of biochemical and anthropometric parameters, liver enzymes, and noninvasive liver fibrosis indices in individuals with T2DM at the study's outset.

**Table 1:** Baseline characteristics of T2DM individuals Data expressed as mean (±SD) and median (interquartile range [IQR]). T2DM: type 2 diabetes mellitus; BMI: body mass index; WC: waist circumference; WHR: waist-to-hip ratio; WtHR: waist-to-height ratio; HDL: high-density lipoprotein; TCh: total cholesterol; SBP: systolic blood pressure; DBP: diastolic blood pressure; AST: aspartate aminotransferase; ALT: alanine aminotransferase; AP: alkaline phosphatase; GGT: gamma-glutamyl transferase; GPR: gamma-glutamyl transpeptidase to platelet ratio; APRI: AST to platelet ratio index; FIB-4: Fibrosis-4; AARPRI: aspartate aminotransferase to alanine aminotransferase ratio to platelet ratio index

Parameters	Value
BMI (kg/m^2^)	24.58 (23.72-25.27)
WC (cm)	93 (86-98.5)
WHR	0.97 (0.95-1.007)
WtHR	0.53 (0.52-0.56)
Fasting glucose (mmol/L)	5.8 (5.6-6)
Triglycerides (mmol/L)	1.5 (1.1-2.07)
HDL (mmol/L)	1.2 (1.1-1.3)
TCh (mmol/L)	4.6 (4.1-5.17)
SBP (mmHg)	130 (130-140)
DBP (mmHg)	90 (80-100)
AST (U/L)	38 (28.25-51.25)
ALT (U/L)	35 (28-39)
AP (U/L)	89 (75.25-97.75)
GGT (U/L)	56.5 (43.25-68)
Platelets (10^9^/L)	315.5 (256.25-348.75)
APRI	0.264 (0.203-0.389)
AGPR	0.467 (0.409-0.528)
AARPRI	0.593 (0.428-0.816)
FIB-4 index	1.008 (0.77-1.504)
FORNS index	6.25 ± 0.75
GPR	0.25 ± 0.084

Based on the MetS development, T2DM individuals with MetS (n=48; 52.5%) showed significantly higher values of APRI (0.425 [0.297-0.481] vs 0.231 [0.171-0.280]; p<0.001), AGPR (0.494 [0.421-0.622] vs 0.45 [0.4-0.51]; p<0.05), AARPRI (0.792 [0.555-0.962] vs. 0.50 [0.34-0.66]; p<0.001), and FIB-4 index (1.503 [1.033-1.861] vs 0.87 [0.69-1.07]; p<0.001) compared to those without MetS (n=32;47.5%) (Table 2).

**Table 2:** Baseline levels of noninvasive liver fibrosis indices in T2DM individuals related to MetS development Data expressed as mean (±SD) and median (the interquartile range-IQR). The student's t-test assessed differences in normally distributed variables, while the Mann-Whitney U test was applied for non-normally distributed variables. T2DM: type 2 diabetes mellitus; MetS: metabolic syndrome; APRI: AST to platelet ratio index; AARPRI: aspartate aminotransferase to alanine aminotransferase ratio to platelet ratio index; AGPR: aspartate aminotransferase to gamma-glutamyl transferase to platelet ratio; GPR: gamma-glutamyl transpeptidase to platelet ratio; FIB-4: Fibrosis-4; *p<0.05; **p<0.001

Parameters	T2DM-MetS (n=48)	T2DM-non-MetS (n=32)	p-value
APRI	0.425 (0.297-0.481)	0.231 (0.171-0.280)	0.000**
AGPR	0.494 (0.421-0.622)	0.45 (0.40-0.51)	0.041*
AARPRI	0.792 (0.555-0.962)	0.50 (0.34-0.66)	0.000**
FIB-4 index	1.503 (1.033-1.861)	0.87 (0.69-1.07)	0.000**
FORNS index	6.46 ± 0.66	6.11 ± 0.77	0.473
GPR	0.27 ± 0.079	0.243 ± 0.087	0.894

**Figure 1:**
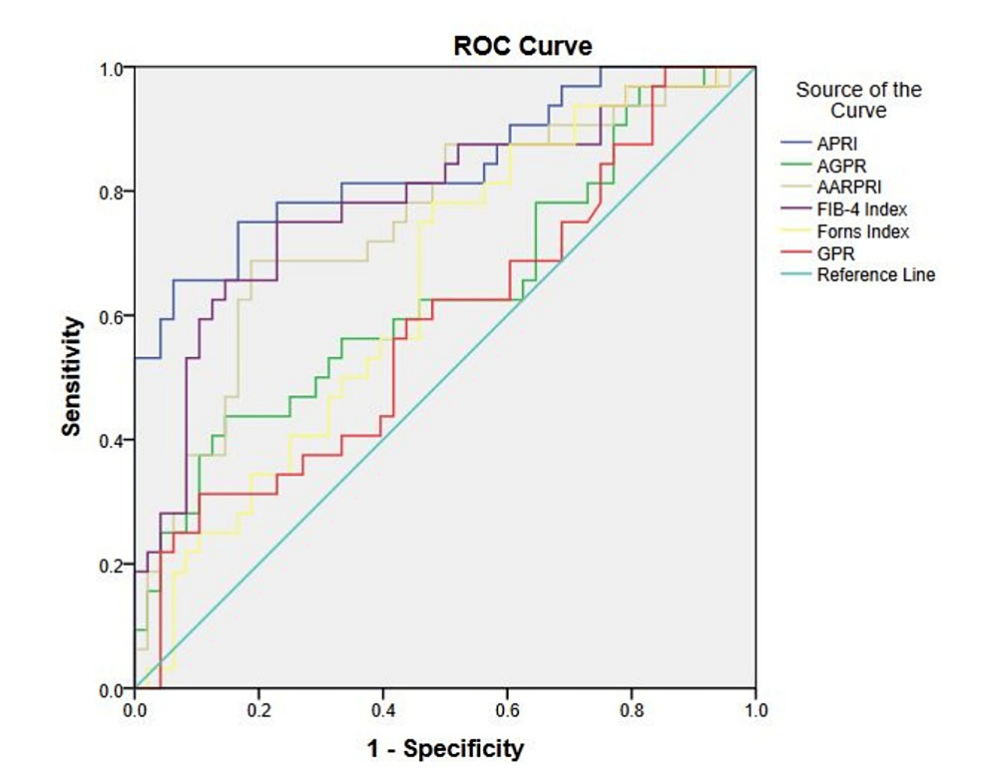
ROC curve analysis for differentiating MetS using noninvasive liver fibrosis indices in T2DM individuals Data expressed with ROC (receiver operating characteristic) analysis. T2DM: type 2 diabetes mellitus; MetS: metabolic syndrome; AARPRI: aspartate aminotransferase to alanine aminotransferase ratio to platelet ratio index; AGPR: aspartate aminotransferase to gamma-glutamyl transferase to platelet ratio; APRI: aspartate aminotransferase to platelet ratio index; FIB-4: Fibrosis-4 index; GPR: gamma-glutamyl transpeptidase to platelet ratio

ROC analysis was utilized to determine the usefulness of noninvasive liver fibrosis indices for diagnosing MetS in individuals with T2DM. The AUC was found to be highest for APRI (0.84), followed by FIB-4 (0.783) and AARPRI (0.747). These findings are illustrated in Figure 1 and displayed in Table 3.

**Table 3:** Assessment of noninvasive liver fibrosis indices for diagnosing MetS in T2DM individuals using ROC analysis APRI: AST to platelet ratio index; AARPRI: aspartate aminotransferase to alanine aminotransferase ratio to platelet ratio index; FIB-4 Fibrosis-4; GPR: gamma-glutamyl transpeptidase to platelet ratio; AUC: area under curve; ROC: receiver operating characteristic; T2DM: type 2 diabetes mellitus; 95% CI: 95% confidence interval; *p<0.05; **p<0.001

	AUC	95% CI	P-value	Cut-off	Sensitivity (%)	Specificity (%)
APRI	0.84	0.746-0.934	0.000**	0.2458	81.3	67.7
AGPR	0.635	0.508-0.763	0.041*	0.4588	62.5	54.4
AARPRI	0.747	0.634-0.859	0.000**	0.5909	71.9	62.5
FIB-4 index	0.783	0.675-0.890	0.000**	0.9317	81.3	56.2
FORNS index	0.643	0.522-0.764	0.031*	0.0056	78.1	52.1
GPR	0.584	0.455-0.713	0.207	0.2417	62.5	50

Analyzing predictors of MetS in T2DM individuals after 24 months of observation, it was found that the APRI was an independent positive predictor of MetS (OR 18.179, 95% CI 6.035-24.58, p=0.015) (Table 4).

**Table 4:** The logistic regression analysis predicting MetS in T2DM individuals APRI: aspartate aminotransferase to platelet ratio index; AGPR: aspartate aminotransferase to gamma-glutamyl transferase to platelet ratio; AARPRI: aspartate aminotransferase to alanine aminotransferase ratio to platelet ratio index; GPR: gamma-glutamyl transpeptidase to platelet ratio; FIB-4: Fibrosis-4; OR: odds ratio; CI: confidence interval; T2DM: type 2 diabetes mellitus; MetS: metabolic syndrome; *p<0.05

Model	B	Standard error	P-value	Exp(B)	95% CI
APRI	9.408	3.883	0.015*	18.179	6.035-24.58
AGPR	0.824	5.099	0.872	2.281	0.041-4.9
AARPRI	-4.786	2.877	0.096	0.008	0.012-2.23
FIB-4 Index	4.337	1.991	0.059	6.445	0.944-3.78
FORNS Index	-1.401	0.793	0.077	0.246	0.052-1.16
GPR	7.586	6.319	0.230	1.969	0.008-4.709
Dependent Variable: Metabolic Syndrome after 24 months of observation					

## Discussion

To our knowledge, this study is the first prospective investigation exploring whether non-invasive liver fibrosis indices, such as the APRI, AARPRI, AGPR, and FIB-4, can serve as markers and predictors of MetS development in T2DM individuals. The results demonstrated that APRI, AARPRI, AGPR, and FIB-4 are good markers for distinguishing T2DM individuals with and without MetS based on AUC values through ROC. Additionally, binary logistic regression identified APRI as an independent positive predictor of MetS development in T2DM.

The mechanisms linking MetS, obesity, T2DM, NAFLD, and liver fibrosis are complex and multifaceted, involving genetic and environmental factors that disrupt energy metabolism and immune response, leading to dysfunction in various organs, including the liver. Obesity, which is a key risk factor for MetS, also contributes significantly to the development of NAFLD, which can progress to NASH, fibrosis, cirrhosis, and liver cancer [3,18].

NAFLD and T2DM have a bidirectional relationship. Insulin resistance increases liver fat accumulation, while NAFLD heightens the risk of diabetes by disrupting glucose metabolism. Liver fibrosis further complicates insulin resistance and blood sugar control. Nearly 90% of NAFLD patients exhibit multiple MetS features, with insulin resistance driving the flow of free fatty acids to the liver, raising hepatic triglyceride levels, and contributing to NAFLD. It remains unclear whether hepatic insulin resistance causes or results from hepatic steatosis. The accumulation of triacylglycerol and diacylglycerol in fatty livers, along with the activation of protein kinase Cε, hinders liver insulin function. Thus, NAFLD is closely linked to liver insulin resistance, which worsens and contributes to the onset and progression of T2DM [19,20].

NAFLD and MetS are closely linked, potentially causing and resulting from one another. Studies have shown that NAFLD prevalence is higher in individuals with MetS, and all components of MetS are associated with the degree of hepatic steatosis. Gangireddy et al. found that individuals with MetS had over three times the risk of hepatic steatosis and fibrosis compared to those without this syndrome, with MetS being an independent risk factor for liver fibrosis even in the absence of steatosis [21]. Similarly, Shi et al. reported that MetS is common among NAFLD patients. MetS shows a higher incidence of significant fibrosis and elevated liver stiffness measurements (LSM) than those without MetS [22].

The pathogenesis of NAFLD and MetS involves inflammatory processes triggered by the release of cytokines from adipocytes and immune cells. Key cytokines such as tumor necrosis factor-alpha (TNF-α) and interleukin 6 (IL-6) play a crucial role in developing insulin resistance and MetS. Insulin resistance increases the flux of free fatty acids from adipose tissue to the liver, elevating hepatic triglyceride levels and contributing to the development of NAFLD. These processes result in chronic inflammation, which can progress to liver fibrosis [23-25].

Understanding the development of liver fibrosis is crucial as it significantly predicts mortality in metabolic disorders. One primary cause of liver fibrosis is the worsening of hepatocyte steatosis due to increased de novo lipogenesis, which is vital for NAFLD and NASH. Hyperglycemia and high insulin levels drive de novo lipogenesis-hyperglycemia through ChREBP and hyperinsulinemia through SREBP1c. Additionally, high free fatty acid levels, resulting from insulin resistance in adipose tissue, lead to triglyceride formation in hepatocytes. These factors contributing to hepatic steatosis are likely augmented by a direct fibrogenic mechanism, expected to cause fibrosis in multiple organs in T2DM and MetS [26].

Liver enzymes have not consistently been shown to be associated with MetS, highlighting the need to include other factors, such as PLT and lipid profile abnormalities, to improve the prediction and understanding of MetS development. Platelets play a significant role in the pathogenesis of metabolic diseases like MetS and T2DM. Platelet hyperreactivity, a key factor in thrombotic diseases, is commonly observed in diabetes. T2DM patients have higher levels of the collagen receptor glycoprotein VI (GPVI) on platelets, which is associated with cardiovascular events. Glycation of platelet proteins reduces membrane fluidity and increases adhesion, resulting in hyperactive platelets that contribute to thrombotic events [27].

Our study confirms the clinical significance of noninvasive markers such as APRI, AARPRI, AGPR, and FIB-4 as simple, cost-effective tools for assessing liver fibrosis using routine laboratory tests. APRI and FIB-4 are practical alternatives to invasive tests like liver biopsies. APRI considers the AST and platelets and combines two biological phenomena occurring when NAFLD progresses. It is commonly used to evaluate liver fibrosis in hepatitis B or C patients using standard lab tests, while FIB-4 also considers factors like age [28].

Verma et al. found that APRI scores were significantly higher in patients with breast carcinoma with MetS [29]. De Matteis et al. demonstrated that high APRI scores significantly correlate with increased cardiovascular risk, particularly in females, supporting our findings [30]. Solomon et al. concluded that combining noninvasive tests enhances the accuracy of detecting liver involvement in suspected metabolic liver disease, aligning with our results [31].

The clinical implications of our findings are profound, given the increasing prevalence of obesity, MetS, and T2DM globally. Early identification of MetS in T2DM patients using non-invasive markers like APRI, AARPRI, AGPR, and FIB-4 could lead to timely interventions aimed at preventing the progression of liver disease and reducing the risk of associated complications. This approach could significantly impact patient management and outcomes, highlighting the need for routine screening and monitoring of liver health in individuals with T2DM.

Our study has several limitations. First, the sample size was relatively small, limiting the generalizability of our findings and highlighting the need for larger studies to validate our results. Second, the lack of similar studies in the literature made it challenging to compare our results directly with those of other researchers. This underscores the novelty of our study and the necessity for further research in this area to confirm and expand upon our findings.

## Conclusions

Our study adds valuable evidence to the existing body of knowledge about the role of non-invasive liver fibrosis markers in predicting MetS in T2DM patients. By highlighting the utility of APRI, we pave the way for more comprehensive and proactive management strategies for patients at risk of MetS and its complications. Further research is needed to confirm our findings and explore additional potential non-invasive markers. Longitudinal studies tracking patients over time could provide more information on long-term outcomes and the effectiveness of early interventions based on identified markers. Additionally, understanding the molecular mechanisms underlying the relationships between obesity, T2DM, MetS, NAFLD, and liver fibrosis will be crucial for developing targeted therapies and improving patient care.
